# Supplementary figures and images for: Combination administration of alprazolam and N-Ethylmaleimide synergistically enhances sleep behaviors in mice with no potential CNS side effects
Source: PeerJ. 2024 May 7;12:e17342. doi: 10.7717/peerj.17342 (PMC11086308; doi:10.7717/peerj.17342)

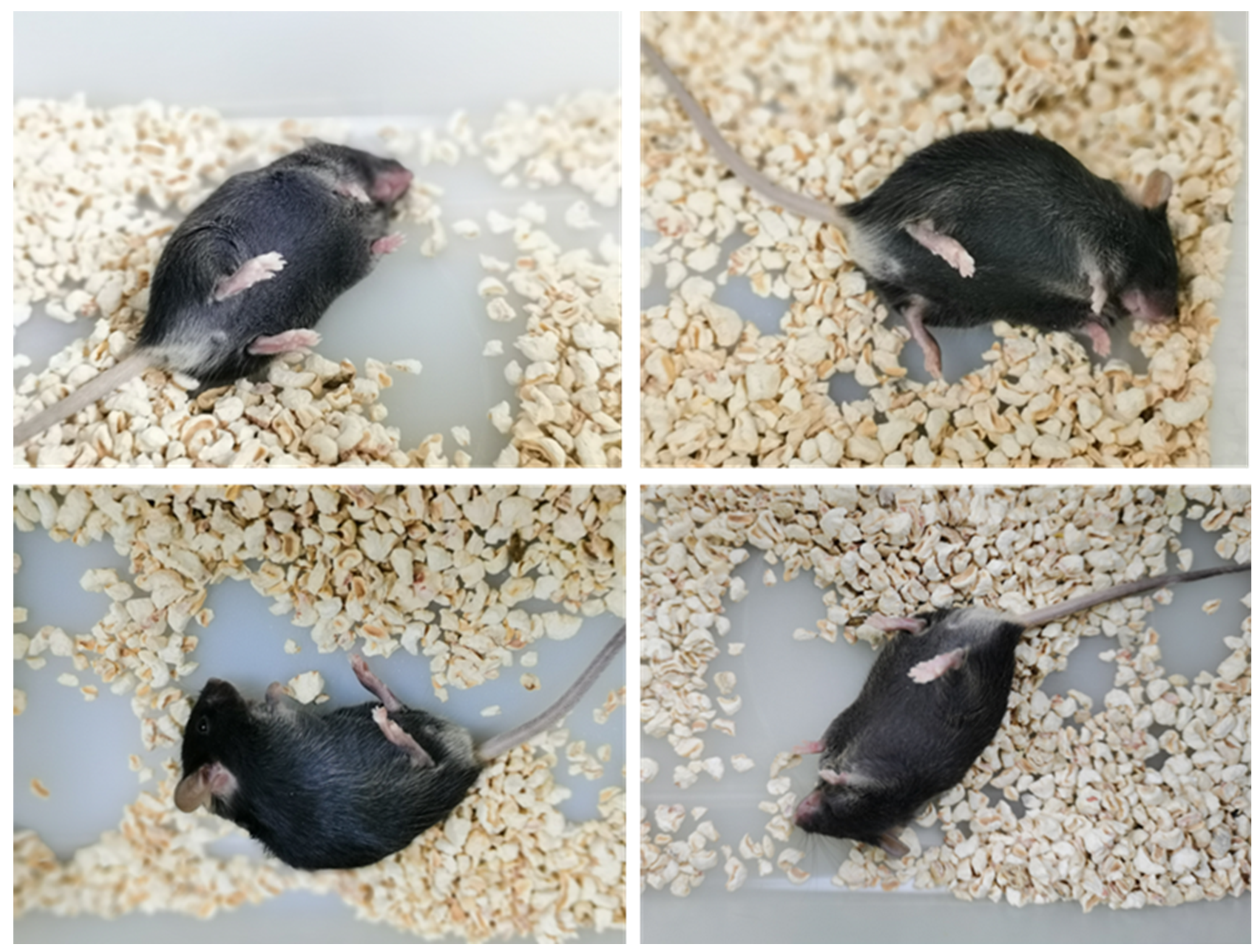

Supplement: Figure S1 [file peerj-12-17342-s001.png]

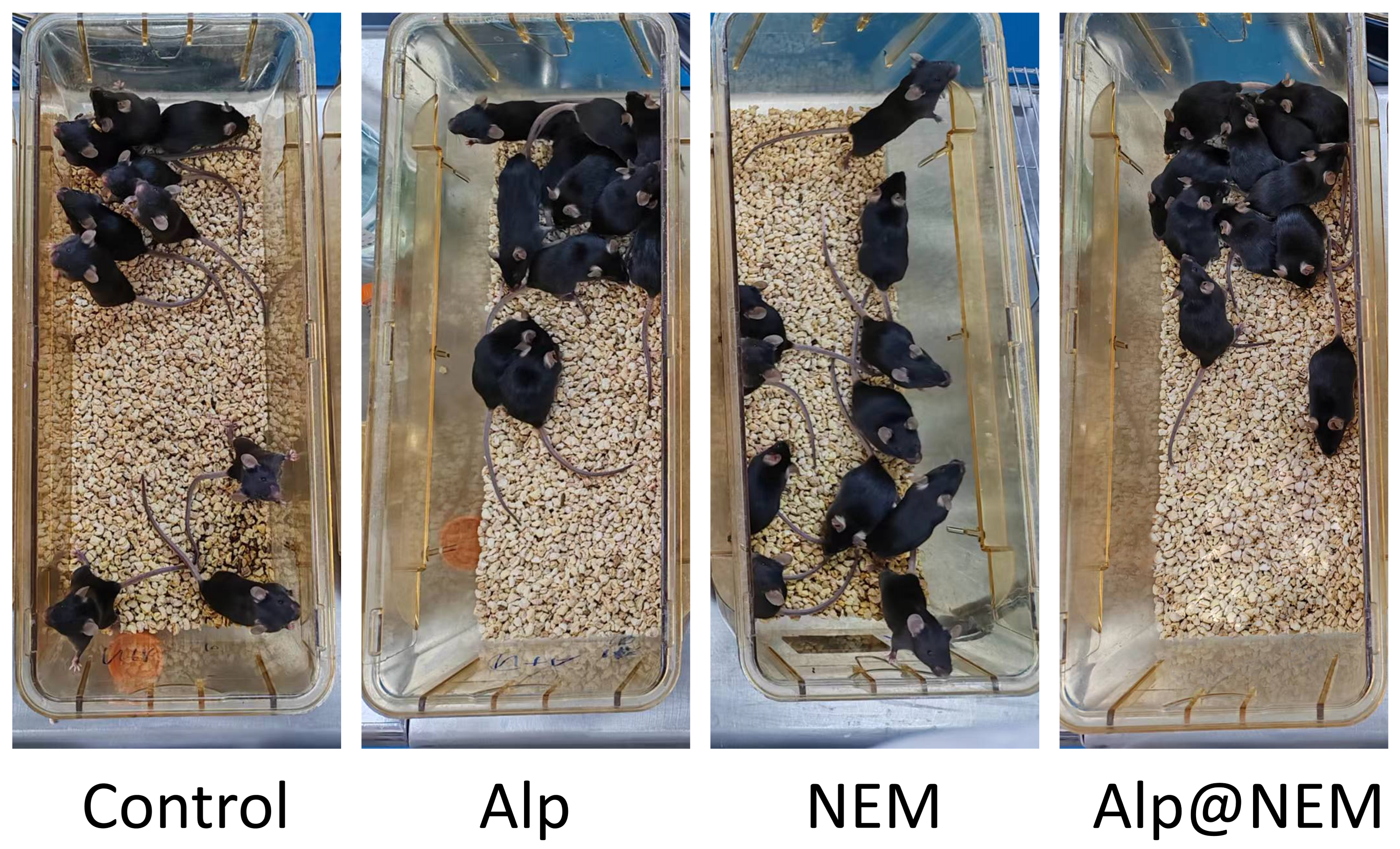

Supplement: Figure S2 [file peerj-12-17342-s002.png]

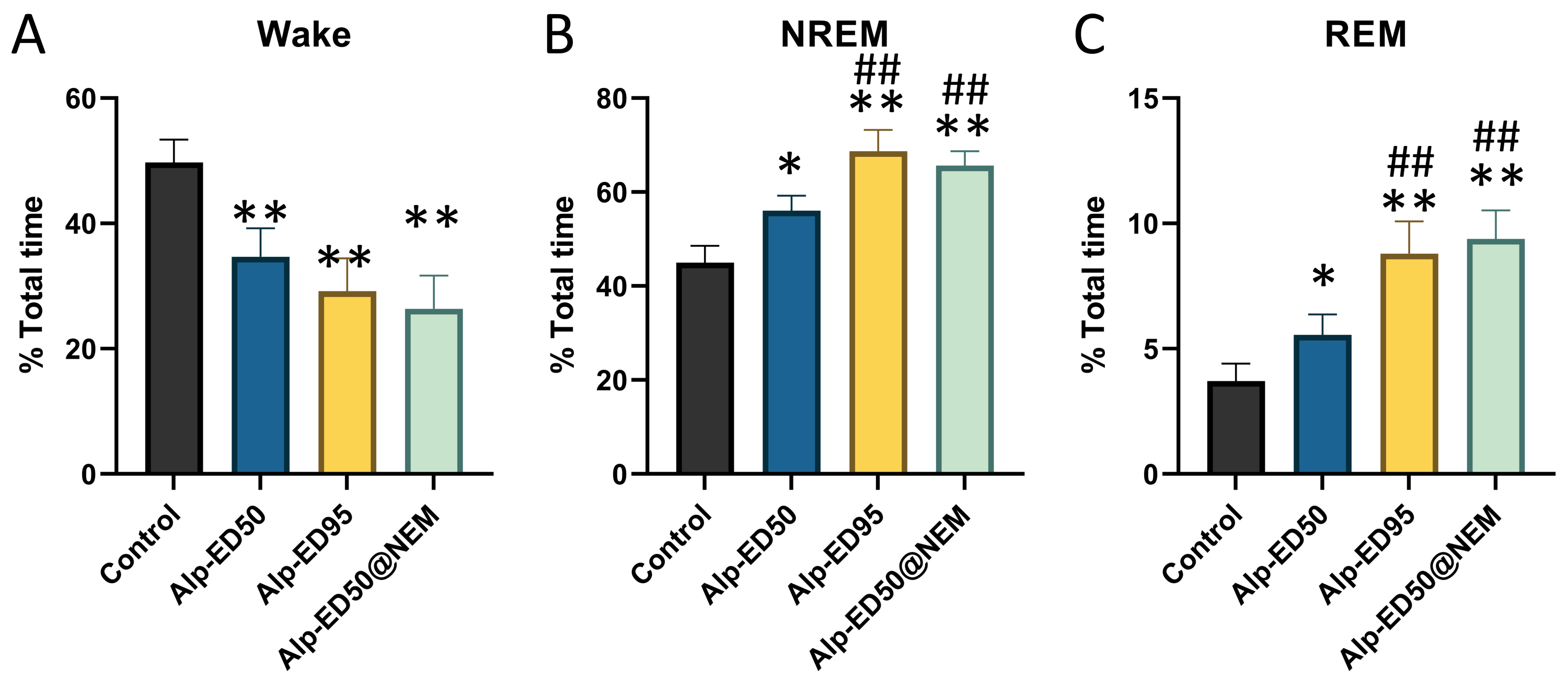

Supplement: Figure S3 — (A) wake, (B) NREM and (C) REM in the total time of the mice after treatment. Data were expressed as mean ± SEM (n = 8). *p < 0.05 and **p < 0.01 vs. control. # p < 0.05 vs. Alp-ED_50 [file peerj-12-17342-s003.png]
